# Correction: Suppression of Mitochondrial Electron Transport Chain Function in the Hypoxic Human Placenta: A Role for miRNA-210 and Protein Synthesis Inhibition

**DOI:** 10.1371/journal.pone.0093245

**Published:** 2014-04-04

**Authors:** The PLOS ONE Staff

**Notice of Republication**

This article was republished on February 7, 2014, to update the figure legends for Figures 2-4, which were incorrectly labeled in the original version of the article. Please download this article again to view the correct version. The originally published, uncorrected article and the republished, corrected article are provided here for reference.

## Supporting Information

File S1 Originally published, uncorrected article.

File S2 Republished corrected article.
