# Supplementary figures and images for: Suppression of Mitochondrial Electron Transport Chain Function in the Hypoxic Human Placenta: A Role for miRNA-210 and Protein Synthesis Inhibition
Source: PLoS One. 2013 Jan 30;8(1):e55194. doi: 10.1371/journal.pone.0055194 (PMC3559344; doi:10.1371/journal.pone.0055194)

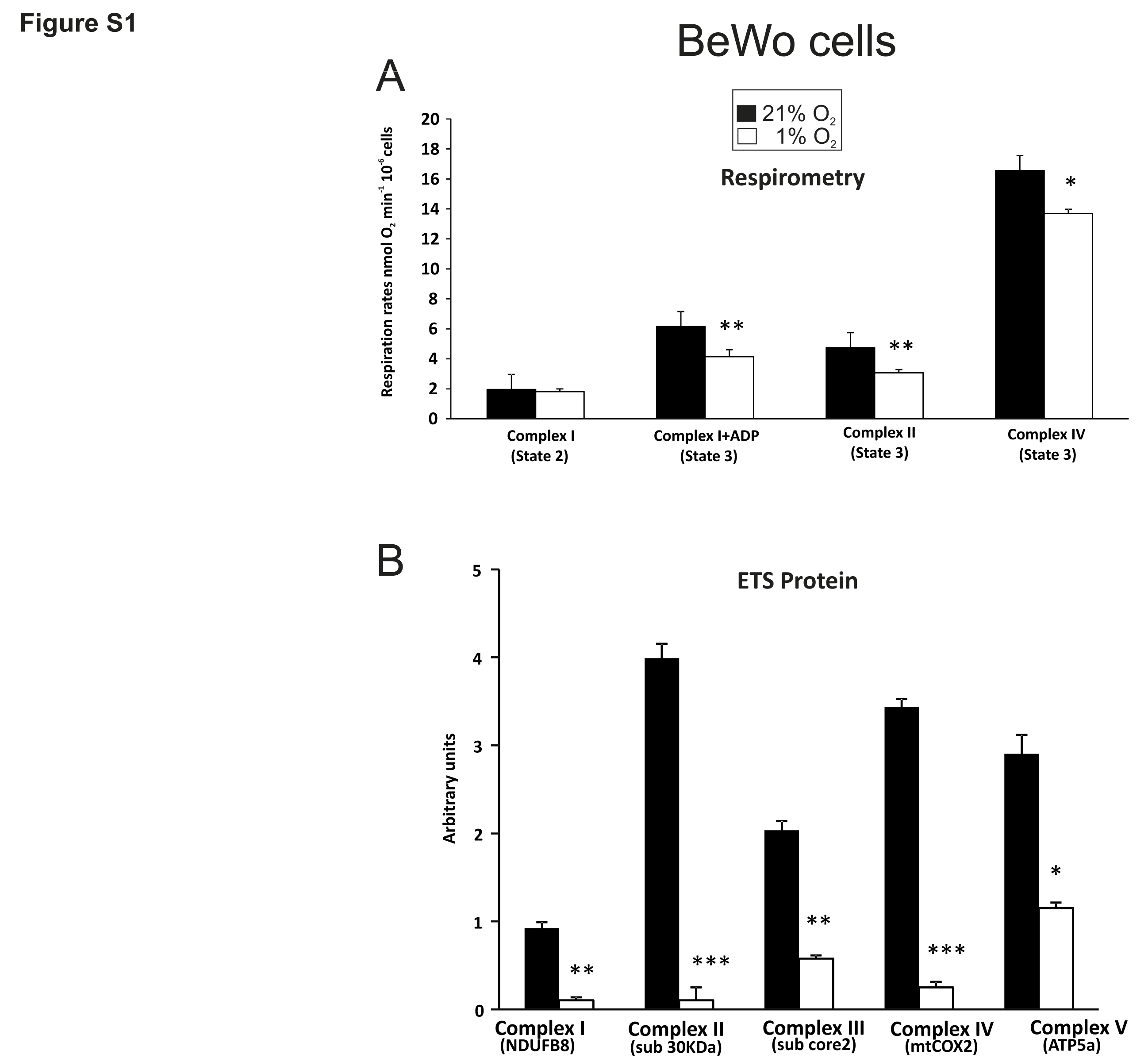

Supplement: Figure S1 — Mitochondrial respiratory function and ETS protein expression were altered in BeWo cells cultured in hypoxic conditions. A) State 2 and state 3 respiration rates with the complex I substrates, glutamate and malate; and state 3 respiration rates with the complex II substrate, succinate, and complex IV substrates, TMPD and ascorbate in BeWo cells. B) Protein levels of ETS complexes I–IV and V (ATP-synthase) in BeWo cells. Three independent experiments were performed in duplicate for each condition; *p<0.05, **p<0.01, ***p<0.001 compared with cells cultured at 21% O2. (TIF) [file pone.0055194.s001.tif]

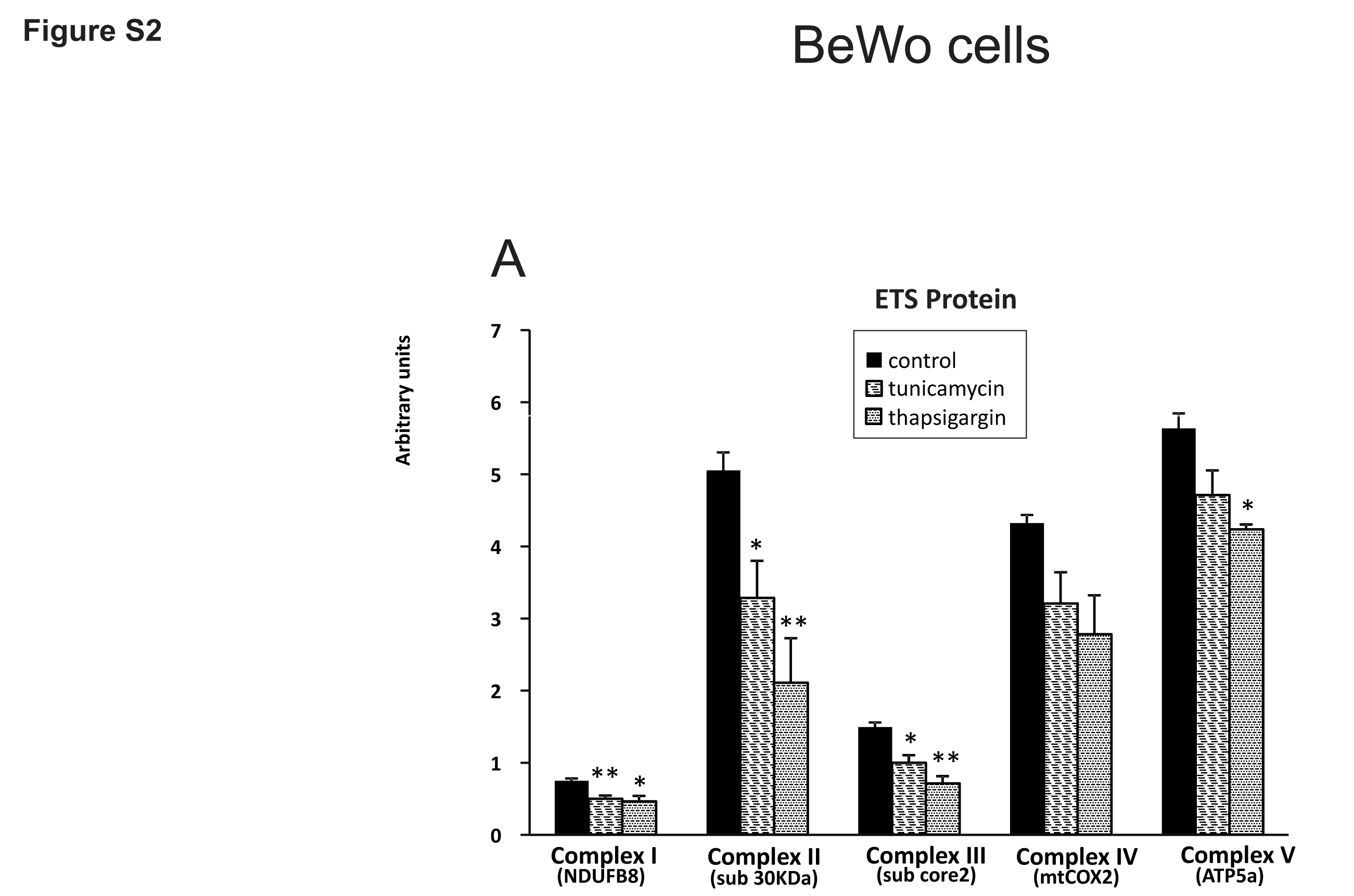

Supplement: Figure S2 — Treatment with a sublethal dose of tunicamycin (2.5 µg/ml) or thapsigargin (0.4 µM) downregulates ETS protein levels in BeWo cells. A) Protein levels of ETS complexes I–IV and V (ATP-synthase) in BeWo cells. *p<0.05, **p<0.01 compared with cells cultured without tunicamycin or thapsigargin. Three biological replicates for control and treated cells were performed. (TIF) [file pone.0055194.s002.tif]
