# Supplementary material for: Correction: Suppression of Mitochondrial Electron Transport Chain Function in the Hypoxic Human Placenta: A Role for miRNA-210 and Protein Synthesis Inhibition
Source: PLoS One. 2014 Apr 4;9(4):e93245. doi: 10.1371/journal.pone.0093245 (PMC3976266; doi:10.1371/journal.pone.0093245)
Supplement: File S1 — Originally published, uncorrected article. [file pone.0093245.s001.pdf]

OPEN ACCESS Freely available online PLOS | ONE

Suppression of Mitochondrial Electron Transport Chain Function in the Hypoxic Human Placenta: A Role for Mirna-210 and Protein Synthesis Inhibition

Francesca Colleoni[1]*, Nisha Padmanabhan[1]◑, Hong-wa Yung[1]◑, Erica D. Watson[1], Irene Cetin[2], Martha C. T i s s o t van Patot[1], Graham J. Burton[1], Andrew J. Murray[1]

1 Department of Physiology, Development & Neuroscience, and Centre for Trophoblast Research, University of Cambridge, Cambridge, United Kingdom, 2 Unit of Obstetrics and Gynecology, Department of Clinical Sciences "Luigi Sacco" University of Milan, Milan, Italy

Abstract

Fetal growth is critically dependent on energy metabolism in the placenta, which drives active exchange of nutrients. Placental oxygen levels are therefore vital, and chronic hypoxia during pregnancy impairs fetal growth. Here we tested the hypothesis that placental hypoxia alters mitochondrial electron transport chain (ETS) function, and sought to identify underlying mechanisms. We cultured human placental cells under different oxygen concentrations. Mitochondrial respiration was measured, alongside levels of ETS complexes. Additionally, we studied placentas from sea-level and high-altitude pregnancies. After 4 d at 1% O_2 (1.01 KPa), complex I-supported respiration was 57% and 37% lower, in trophoblast-like JEG3 cells and fibroblasts, respectively, compared with controls cultured at 21% O_2 (21.24 KPa); complex IV-supported respiration was 22% and 30% lower. Correspondingly, complex I levels were 45% lower in placentas from high-altitude pregnancies than those from sea-level pregnancies. Expression of HIF-responsive microRNA-210 was increased in hypoxic fibroblasts and high-altitude placentas, whilst expression of its targets, iron-sulfur cluster scaffold (ISCU) and cytochrome *c* oxidase assembly protein (COX10), decreased. Moreover, protein synthesis inhibition, a feature of the high-altitude placenta, also suppressed ETS complex protein levels. Our results demonstrate that mitochondrial function is altered in hypoxic human placentas, with specific suppression of complexes I and IV compromising energy metabolism and potentially contributing to impaired fetal growth.

Citation: Colleoni F, Padmanabhan N, Yung H-w, Watson ED, Cetin I, et al. (2013) Suppression of Mitochondrial Electron Transport Chain Function in the Hypoxic Human Placenta: A Role for Mirna-210 and Protein Synthesis Inhibition. PLoS ONE 8(1): e55194. doi:10.1371/journal.pone.0055194

Editor: Yidong Bai, University of Texas Health Science Center at San Antonio, United States of America

Received August 2, 2012; **Accepted** December 19, 2012; **Published** January 30, 2013

Copyright: © 2013 Colleoni et al. This is an open-access article distributed under the terms of the Creative Commons Attribution License, which permits unrestricted use, distribution, and reproduction in any medium, provided the original author and source are credited.

Funding: Dr Murray thanks the Research Councils UK for supporting his academic fellowship and Prof Burton gratefully acknowledges support for his research from the Wellcome Trust (084804/2/08/Z). This study was primarily supported by Action Medical Research, project grant number SP4545. The funders had no role in study design, data collection and analysis, decision to publish, or preparation of the manuscript.

Competing Interests: The authors have declared that no competing interests exist.

* E-mail: fc316@cam.ac.uk

◑ These authors contributed equally to this work.

Introduction

During the first trimester of pregnancy, the human fetus develops in an environment characterised by a very low partial pressure of oxygen (pO_2) [1], which is strikingly close to that experienced by mountaineers high on Mt Everest [2]. This condition was termed Everest *in utero*, by Joseph Barcroft more than 60 years ago, and is believed to favour organogenesis in the embryo, and cell proliferation and angiogenesis in the placenta [1].

At high altitude, where women are exposed to atmospheric hypobaric hypoxia, the hypoxic nature of the uterine environment is further exacerbated.Such chronic exposure to hypobaric hypoxia during pregnancy leads to babies that are small for gestational age [3], and an increased incidence of intrauterine growth restriction and pre-eclampsia [4,5]. Curiously, normal oxygen delivery to the fetus is maintained at altitudes of around 3000 m above sea-level despite a lower arterial partial pressure of oxygen, at least in part due to increased erythropoiesis in both the maternal and fetal circulations [6]. Somewhat paradoxically, however, fetal oxygen consumption at high-altitude, when corrected for body weight, is not different to that at sea level [7], thus oxygen deprivation *per se* is not thought to be responsible for the impairment in fetal growth. Instead, it has been proposed that the high altitude placenta undergoes metabolic remodelling to lower its own oxygen consumption, thereby maintaining oxygen delivery to the fetus but at the cost of altered substrate delivery. This concept has been extensively reviewed by ourselves and others [8–9], yet the underlying mechanisms remain unresolved.

Placental dysfunction lies at the core of many common complications of pregnancy, such as intrauterine growth restriction and pre-eclampsia. These disorders can jeopardise the health of both mother and fetus, accounting for ~60% of babies weighing less than 1000 g that survive to only one year of life [10]. The pathophysiology of pre-eclampsia is not completely understood, but is thought to result from incomplete remodelling of the maternal spiral arteries [11], disrupting the normal flow of blood into the placenta and risking ischemia/reperfusion injury [1]. Indeed, the induction of oxidative stress is a component of pre-

eclampsia [12], supported by reports of increased pro-oxidant factors [13,14] and decreased anti-oxidant defences [14].

In this regard, placental mitochondria are likely to play a central role in pre-eclampsia, being producers of reactive oxygen species (ROS) at complexes I and III of the electron transport system (ETS), and themselves targets of oxidative stress. Increased superoxide production has been reported in pre-eclamptic placentas [15], suggesting that the mitochondria are at increased risk of oxidative damage. Indeed, in other metabolically-active tissues, such as cardiac and skeletal muscle, oxidative stress is associated with profoundly altered mitochondrial function [16,17]. For example, in hypoxic skeletal muscle, the downregulation of ETS complexes I and IV may be an adaptive response to respectively limit ROS production and oxygen consumption [18]. Additionally, recent data suggest that in early-onset pre-eclampsia there is a high incidence of endoplasmic reticulum (ER) stress, a phenomenon strongly associated with oxidative stress and which shares a similar etiology [19].

Stabilization of hypoxia-inducible factor-1α (HIF-1α) under hypoxic conditions leads to a downregulation of mitochondrial oxygen consumption [20,21], and the HIF-responsive microRNA-210 (miR-210) has been strongly implicated in this response [22,23]. MiR-210 represses the iron-sulfur complex assembly proteins (ISCU1/2) [23], which are required for the correct assembly of iron-sulfur clusters in ETS complexes I, II and III. It also represses the cytochrome *c* oxidase assembly protein (COX10) [24], which is essential for assembly of ETS complexes I and IV. HIF-mediated induction of miR-210 is, therefore, a potential mechanism underlying placental remodelling in the oxidatively-stressed high-altitude placenta, and is known to be elevated in placental tissue derived from pre-eclamptic patients [25,26,27], and in a recent study was shown to regulate trophoblast mitochondrial respiration in pre-eclampsia [27]. An alternative mechanism, however, may result from protein synthesis inhibition, since there is marked evidence of ER stress resulting in protein synthesis inhibition in high-altitude placentas [28], a feature shared with the pre-eclamptic placenta [19]. Protein synthesis inhibition might therefore restrict the synthesis of ETS complex subunits, further repressing oxidative metabolism at the placenta.

In this study, we aimed to determine the effects of chronic hypoxia on mitochondrial function in the human placenta and the underlying mechanisms. We investigated mitochondrial respiration and mRNA and protein expression of ETS complexes in two placental cell types grown at different oxygen tensions; a human trophoblast-like cell line and primary human placental fibroblasts. Volumetrically, these cells represent the principal components of the placenta, but may have different metabolic properties due to their different functions. JEG3 cells possess many biological and biochemical characteristics of syncytiotrophoblasts [29], they produce placental hormones and express enzymes involved in steroidogenesis. They have been widely used to examine the hormonal function of trophoblast cells and intracellular receptor mechanisms, and as a model in a great number of studies devoted to the effects of endocrine-active compounds (xenoestrogens, fitoestrogens, dioxins and pesticides) [29]. We also carried out supporting experiments in cultures of the additional, trophoblast-like BeWo cell line. Meanwhile fibroblasts, in addition to being the most numerous cell type in the placenta, perform the functions of stromal biosynthesis and structural support, and are therefore essential to the growth of the villous trees, itself a metabolically-demanding process.

Furthermore, we measured the expression of miR-210 and its downstream targets, to determine whether they could be implicated in a potential mechanism. Moreover, to investigate whether protein synthesis inhibition suppresses mitochondrial function, we studied the same placental cells when cultured at 21% O_2 in the presence of a non-lethal dose of salubrinal, a phosphatase inhibitor which prevents dephosphorylation of eukaryotic initiation factor 2 subunit α (eIF2α). Finally, we extended our investigation into human placentas from high-altitude pregnancies, comparing expression levels of mitochondrial proteins, miR-210 and its targets with those in placentas from sea-level pregnancies. We hypothesized that overlapping, cell-specific mechanisms drive modifications of ETS activity in the hypoxic placenta to suppress oxidative metabolism.

Materials and Methods

Ethics Statement

Informed, written consent for the use of placental samples to research adaptations to hypoxia was obtained from subjects recruited at St. Vincent's General Hospital in Leadville, CO, USA (3,100 m above sea-level) with the approval of the Colorado Multiple Institutional Review Board (COMIRB Protocol 00–623),and from subjects recruited at the University College Hospital, London, UK (sea-level), with the approval of The University College London Hospitals Committee on the Ethics of Human Research (the Joint UCL/UCLH Ethics Committee, Ref No: 03/0135).

Chemicals and Reagents

All chemicals and tissue culture reagents were purchased from Sigma-Aldrich and Invitrogen Ltd (Paisley, UK) respectively, except where otherwise mentioned. Salubrinal was purchased from Chem-Bridge Corporation (San Diego, USA). The anti-Hu Total OxPhos Complex primary antibody kit was purchased from Invitrogen, anti-ISCU1/2 (FL-142) from Santa Cruz Biotechnology (Insight Biotechnology, Wembley, UK), anti-COX10 from Proteintech (Manchester, UK) and anti-citrate synthase from Alpha Diagnostics (San Antonio, TX, USA).

Cell Cultures

Primary human placental fibroblasts were a gift from Professor Ashley Moffett (University of Cambridge) and were isolated from first and early second trimester placentas with Local Ethical Committee approval [28]. These cells were grown in Dulbecco's Modified Eagle medium (DMEM), supplemented with 5% HI-FBS, penicillin (100 U/ml), streptomycin (100 μg/ml), at 37°C in a 5% carbon dioxide (CO_2) atmosphere. JEG-3 cells were a gift from Professor Ashley Moffett (University of Cambridge), and were originally purchased from the American Type Culture Collection and cultured according to their instructions (ATCC, cat no. HTB-144 and HTB-36) [28,30]. JEG3 cells were grown in RPMI 1640 medium supplemented with 5% heat-inactivated FBS (HI-FBS), penicillin (100 U/ml), and streptomycin (100 μg/ml) at 37°C in a 5% CO_2 atmosphere. The human choriocarcinoma cell line BeWo cells were a gift from Dr. Stephen Charnock-Jones (University of Cambridge) (ATCC, cat no. CCL-98). [28]. They were cultured in DMEM/F12 medium supplemented with 10% HI-FBS, penicillin (100 U/ml), streptomycin (100 μg/ml), at 37°C in a 5% carbon dioxide (CO_2) atmosphere.

For hypoxia experiments, placental fibroblasts, JEG3 cells and BeWo cells were seeded at a low density with fresh media (RPMI 1640 medium, DMEM and DMEM/F12, respectively) in the presence of HI-FBS, penicillin and streptomycin and were placed directly into humidified hypoxic chambers (Ex Vivo system, Biospherix Ltd, NY, USA) containing 1% O_2 (1.01 KPa)/5% CO_2 balanced in nitrogen for 4 days. Control cells were incubated

at 21% O_2 (21.24 KPa)/5% CO_2 or 10% O_2 (10.11 KPa)/5% CO_2 under standard culture conditions. A concentration of 21% O_2 was chosen as a first control as this is the normal O_2 concentration to which JEG3 and BeWo cells have adapted, whilst 10% O_2 was used to mimic intraplacental conditions [1,10] at the start of the second trimester *in vivo*. For hypoxic conditions, we selected 1% O_2 as this was shown to elicit a hypoxic response in pilot studies. After 4 d, cells were harvested for analysis of mitochondrial respiration, protein, mRNA and miRNA expression levels or mitochondrial DNA (mtDNA) concentrations. After 4 days, hypoxia (1% O_2) decreases the proliferation of JEG3 cells, BeWo cells and placental fibroblasts by 40%, 60% and 18%, respectively [28].

For protein synthesis inhibition experiments, placental fibroblasts and JEG3 cells were seeded at a low density with fresh media as before. Untreated control cells and cells grown with a sublethal dose of salubrinal (17.5 μM) were incubated at 21% O_2/5% CO_2 for 3 d under standard conditions, and harvested for analysis of protein levels. Inhibition of protein synthesis using these cultured conditions was recently confirmed in a study published by our group [28]. BeWo cells were seeded at a low density with fresh media as before. Untreated control cells and cells grown with a sublethal dosage of tunicamycin (2.5 ug/ml) or thapsigargin (0.4 μM) were incubated at 21% O_2/5% CO_2 for 3 d under standard conditions, and harvested for analysis of ETS protein levels.

Tissue Collection from Human Subjects

Exclusion criteria for subjects included renal disease, cardiac disease, diabetes, chronic hypertension, pregnancy-induced hypertension, pre-term delivery or any complication of pregnancy. Placentas (n = 6) were collected at sea level from elective non-labored caesarean deliveries (two tissue samples from different regions of each placenta), whilst 3 placentas (four tissue samples from different regions of each placenta) was collected at 3100 m altitude, again from elective non-labored caesarean deliveries. Since the labor process is a strong inducer of oxidative stress [31], only samples delivered by caesarean section were used in this study. The samples were collected immediately after delivery, by the same team at each site to eliminate differences in tissue handling. Each placenta was weighed and samples were taken using a systematic random system by which each placenta was divided into five areas. Two full-thickness samples were taken from each area. Samples were washed in phosphate buffered saline (PBS) to remove blood, snap-frozen in liquid nitrogen within 10 min of delivery and stored at −80°C until further analysis.

Mitochondrial Respirometry

Mitochondrial respiration was measured in permeabilised cells, as described previously [1,32]. Briefly, harvested cells were washed with PBS and re-suspended in respiratory medium (0.5 mM EGTA, 3 mM $MgCl_2.6H_2O$, 20 mM taurine, 10 mM KH_2PO_4, 20 mM HEPES, 1 mg/ml BSA, 60 mM potassium-lactobionate, 110 mM mannitol, 0.3 mM dithiothreitol, pH 7.1). Density of cell suspensions was determined using a haemocytometer and 2×10^6 cells were added to a final volume of 500 μl respiratory medium, equilibrated to atmospheric O_2, in a water-jacketed oxygen electrode chamber at 37°C (Strathkelvin Instruments Ltd, Glasgow, UK) and the chamber was sealed. Cell membranes were selectively permeabilised with digitonin (50 μg/ml) for 5 min, before mitochondrial respiration was measured.

A substrate/inhibitor titration was used to analyse ETS complexes I, II and IV. Initially, 10 mM glutamate and 5 mM malate were added to the chambers, and complex I-supported state 2 respiration was recorded. State 3 respiration was stimulated by the addition of 2 mM ADP. Next, complex I was inhibited by the addition of 0.5 μM rotenone, before 10 mM succinate was added and complex II-supported state 3 respiration recorded. Electron transport was then inhibited at complex III by addition of 5 μM antimycin. Complex IV-supported state 3 respiration was stimulated by addition of 0.5 mM TMPD and 2 mM ascorbate. Between experiments, oxygen electrode chambers were washed for at least 40 min with 100% ethanol and then several times with water to remove any trace of respiratory inhibitors.

Western Blotting

Cultured cells were harvested and washed with ice-cold PBS, before being scraped into cell lysis buffer (20 mM Tris, 150 mM NaCl, 1 mM EDTA, 1 mM EGTA, 1% Triton X-100, 2.5 mM sodium pyrophosphate, 1 mM glycerolphosphate, 1 mM Na_3VO_4, and complete mini protease inhibitor cocktail (Roche Diagnostics, East Sussex, UK, pH 7.5), and transferred to a microfuge tube. After pipetting up and down ~30 times, cells were kept on ice for 20 min with occasional vortexing and centrifuged at 10,000 g for 5 min. Placental samples were homogenised in the same lysis buffer as the cells using lysing matrix tubes (type D, MP Biomedicals).

Bicinchoninic Acid (BCA) was used to determine protein concentrations in the cultured cell and tissue lysates. Equal amounts of protein were resolved using SDS-PAGE and transferred to nitrocellulose membrane. Western blotting analysis of protein expression was performed as described previously [33] with Ponceau red staining used to normalize for protein-loading. After incubation with primary and secondary antibodies, enhanced chemiluminescence (ECL) (GE Healthcare, Little Chalfont, UK) and X-ray film (Kodak, Hempstead, UK) were used to detect the bands. Band intensity was quantified by ImageJ (U.S. National Institutes of Health, Bethesda, MD, USA).

DNA and RNA Extraction and Quantitative Real-time PCR Analysis

DNA and RNA were extracted from cultured cells and human placental samples using SIGMA GenElute™ Mammalian Genomic DNA Miniprep Kit and QIAGEN RNAeasy Mini kit, respectively according to the manufacturer's instructions. RNA was further treated with TURBO DNase (Ambion) to eliminate DNA contamination. A QIAGEN MiRNeasy Mini Kit was used to extract miRNAs.

For total RNA analysis, cDNA was prepared using the first strand synthesis kit from Fermentas. Real-time PCR was performed using SYBR green Master Mix (Eurogentech) on ABI PRISM 7500 Sequence Detection System. Samples were analysed in triplicate and expression levels were normalised to the housekeeping gene hypoxanthine guanine phosphoribosyl transferase (HPRT) or to HPRT and β-actin, together. Melting curve analysis was performed to ensure specificity of PCR products. Fold change was calculated using a standard curve method. Primer sequences are available upon request.

For microRNA analysis, cDNA was prepared using RevertAid H Minus Reverse Transcriptase (Fermentas) and microRNA specific RT primers (TaqMan MiRNA assay). Real-time PCR of miRNA targets was performed using TaqMan MicroRNA assays (hsa-miR-210) according to manufacturer's instructions and normalised to a reference miRNA (RNU48). Fold change was calculated using ΔΔCt method.

Real-time Quantitative PCR Analysis for Mitochondrial DNA Content

Total DNA was extracted using QIAamp DNA Mini kit Q (Qiagen, Milan, Italy). ABI Prism 7500 Sequence Detection System was used for real-time quantitative polymerase chain reaction (PCR) analysis using the Rnase P gene, as an endogenous control and cytochrome b as mitochondrial target gene. Samples were analysed in triplicate. The Δ cycle threshold (ΔCt) values from each sample were obtained by subtracting the values for the reference gene from the sample Ct, thus normalizing to nuclear DNA.

Statistics

Results are expressed as means ± SEM. All data were checked for normal distribution. Analysis of variance (one way ANOVA) with repeated measures and least significant difference (LSD) *post hoc* independent unpaired *t* tests were used to determine differences between groups in different oxygen environments for JEG3, fibroblasts and BeWo cells (for respirometry, protein and DNA levels). Independent *t* tests were used to determine differences between placental samples from different altitudes and for JEG3 and fibroblasts mRNA quantification experiments. Data were considered statistically significant at $p<0.05$.

Results

Mitochondrial Respiration Rates are Lower in Hypoxic Placental Cells

To investigate whether mitochondrial function was altered in hypoxic placental cells, we first compared respiration in primary human placental fibroblasts cultured under 21% O_2 and hypoxic (1% O_2) conditions. In order to study responses across a broad spectrum of oxygen concentrations, we used three different conditions: 1% O_2, 10% O_2 and 21% O_2. All state 2 and state 3 rates were the same between 21% and 10% O_2 culture conditions indicating that these concentrations could be used as our controls (Figure 1A). In hypoxic conditions, at 1% O_2, state 2 respiration rates of fibroblasts were 56% lower compared with those cultured at 21% O_2 (56% of control rates; $p<0.01$) and 47% lower compared to 10% O_2 (Figure 1A). Complex I-supported state 3 rates were 43% and 36% lower in fibroblasts cultured at 1% O_2 compared with those cultured at 10% O_2 ($p<0.05$) and 21% O_2, respectively (Figure 1A), and there was no significant difference in respiratory control ratios (RCRs; state 3/state 2) between difference culture conditions, suggesting no alteration in proton leak. Complex II-supported state 3 respiratory rates were the same in fibroblasts cultured under all three oxygen conditions (Figure 1A). However, complex IV-supported state 3 rates were 29% and 24% lower in fibroblasts cultured at 1% O_2 ($p<0.01$) compared with those cultured at 21% and 10% O_2, respectively (Figure 1A).

Primary trophoblast cells isolated from human placentas were not suitable for these metabolic studies as they display molecular evidence of ER stress (data not shown). This may account for the fact that mitochondrial membrane potential declines progressively in culture, to the point that it is almost totally lost at 96 h [34]. Over the same period there is activation of caspases 3 and 9, and evidence of apoptotic cell death. Therefore, we analyzed a human placental trophoblast-like cell line (JEG3) to determine whether the hypoxia-induced mitochondrial respiratory rate changes we observed in the placental fibroblasts were cell type-specific. As with primary human placental fibroblasts, there were no differences in all respiratory rates between cells cultured at 21% and 10%. In JEG3 cells cultured at 1% O_2, however, respiration rates were 33% lower compared with cells at 21% O_2 ($p<0.01$), and 22% lower than cells at 10% O_2 ($p<0.05$) (Figure 1B). Complex I-supported state 3 rates were 56% and 52% lower in JEG3 cells cultured at 1% O_2 compared with cells cultured at 21% O_2 ($p<0.05$) and 10% O_2 ($p<0.05$), respectively (Figure 1B) once again, there was no significant difference in RCRs between difference culture conditions, suggesting no alteration in proton leak. Similar to our observation in fibroblasts, no differences were apparent with respect to complex II-supported state 3 respiration rates in JEG3 cells cultured under all three oxygen conditions (Figure 1B). However, complex IV-supported state 3 respiration rates were 22% and 23% lower in cells cultured at 1% O_2 compared with cells cultured at 21% ($p<0.001$) and 10% O_2, respectively ($p<0.001$) (Figure 1B). Together, these data indicate a strikingly similar response of lowered mitochondrial respiration rates under severe hypoxia in two different placental cell types. Supporting experiments were also performed on another chorio-carcinoma cell line, BeWo cells, and demonstrated that hypoxia impaired Complexes I–II and IV-supported state 3 rates (Figure S1A).

Hypoxia Alters the Expression of Electron Transport Chain Complexes in Placental Cells

Since the mitochondrial respiratory rates through complex I were lowered in hypoxic conditions, we sought to determine whether this was due to changes in the expression of components that make up the ETS machinery. Decreased complex I-supported rates can result from a decrease in mitochondrial membrane potential, since many substrates for complex I rely on the proton gradient for import. First, we used qRT-PCR to determine mRNA expression levels of *NDUFB8* (NADH dehydrogenase (ubiquinone) 1 beta subcomplex 8; encodes a complex I subunit), *mtCOX2* (mitochondrially encoded cytochrome *c* oxidase II, also known as MTCO2 or COX2; encodes a complex IV subunit) and *ATP5a* (ATP-synthase 5a; encodes a complex V subunit) in both placental fibroblast and JEG3 cells cultured in hypoxic conditions compared to those cultured in normoxia. Transcript levels of *NDUFB8* were unchanged in both fibroblasts and JEG3 irrespective of O_2 concentration (Figures 1C and 1D). However, mRNA expression levels of *mtCOX2* were 37% ($p<0.05$) and 44% ($p=0.07$) lower in fibroblasts cultured at 1% O_2 compared to fibroblasts at 10% O_2 and 21% O_2, respectively (Figure 1C). *ATP5A* (complex V) mRNA expression was also significantly reduced in fibroblasts grown at 1% O_2 compared to 21% O_2 (49% of control; $p<0.05$) but again did not change in JEG3. Whilst there were changes in mRNA expression in fibroblasts, there were clearly none present in JEG3 cells.

Therefore, to determine whether a post-transcriptional mechanism was operating to regulate the protein subunits of the ETS complexes I–IV (NDUFB8, subunit 30 KDa, subunit core2 and mtCOX2, respectively) and V (ATP5a) in hypoxia, we used western blot analysis to quantify the expression of these proteins. Similar to respiratory rates, protein levels of all subunits tested were unchanged in placental fibroblasts and JEG3 cultured at 21% O_2 and 10% O_2. Although protein levels of the complex I subunit (NDUFB8) in fibroblasts at 1% O_2 was 66% lower compared to 21% O_2 and 72% lower compared to 10% O_2, these values did not reach statistical significance (Figure 1E). Interestingly, mtCOX2, the protein subunit of complex IV showed a 70% decrease in expression in fibroblasts cultured at 1% O_2 compared with fibroblasts cultured at 21% O_2 ($p<0.05$) and 10% O_2 ($p<0.05$) (Figure 1E). This was consistent with a decrease in *mtCOX2* mRNA expression in these cells.

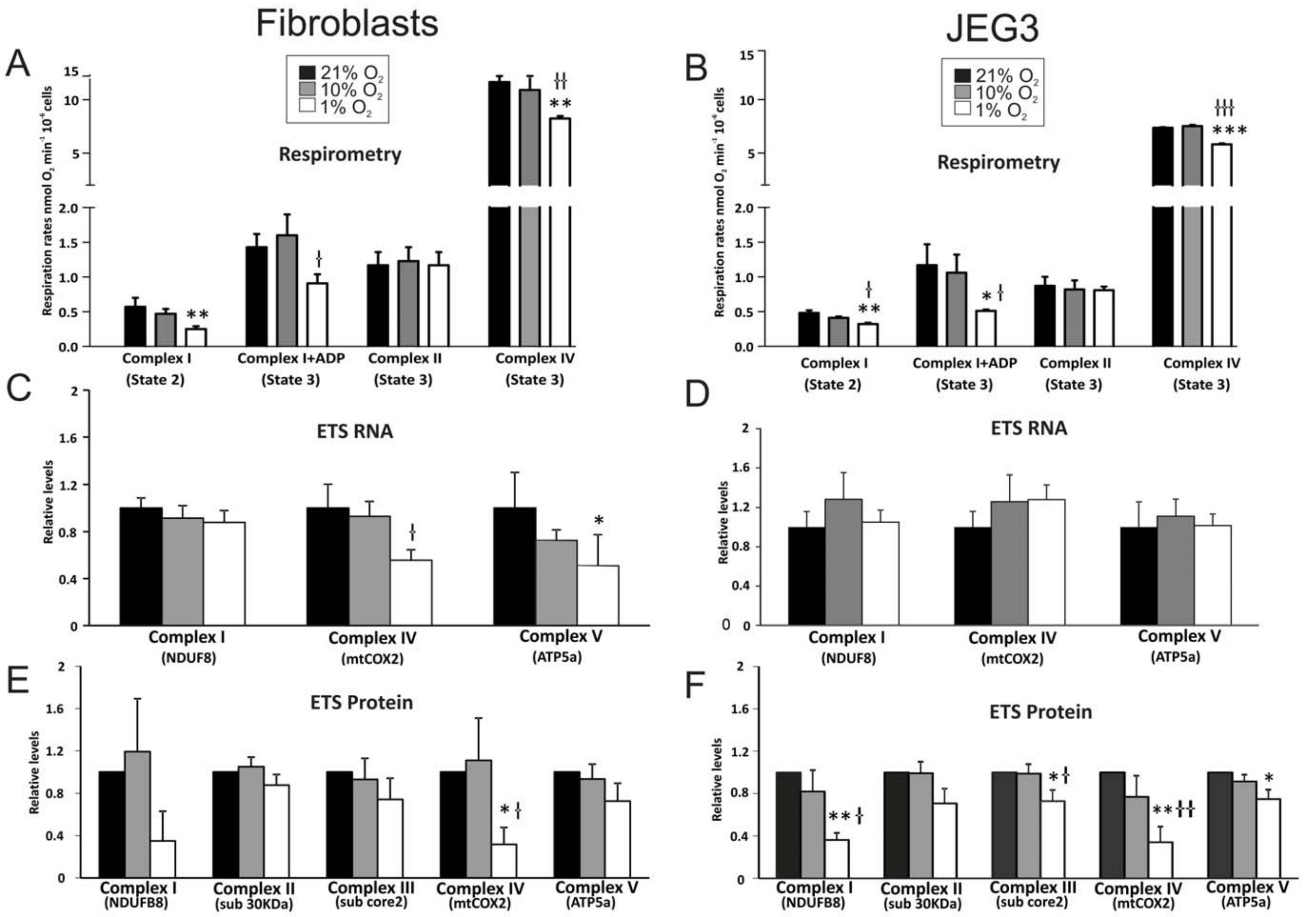

Figure 1. Mitochondrial function and ETS mRNA and protein expression were altered in fibroblasts and JEG3 cells cultured in hypoxic conditions. ***A, B*****)** State 2 and state 3 respiration rates with the complex I substrates, glutamate and malate; and state 3 respiration rates with the complex II substrate, succinate, and complex IV substrates, TMPD and ascorbate in ***A*****)** fibroblasts and ***B*****)** JEG3. ***C, D*****)** Transcript levels of ETS complexes I, IV and V (ATP-synthase) in ***C*****)** fibroblasts and ***D*****)** JEG3. ***E, F*****)** Protein levels of ETS complexes I-IV and V (ATP-synthase) in ***E*****)** fibroblasts and ***F*****)** JEG3. * $p<0.05$, ** $p<0.01$, *** $p<0.001$ compared with cells cultured at 21% O2; † $p<0.05$, †† $p<0.01$, ††† $p<0.001$ compared with cells cultured at 10% O2. Three independent experiments were performed in duplicate for each condition; each experiment was carried out in duplicate.
doi:10.1371/journal.pone.0055194.g001

Similar to the fibroblast cells, levels of complex I subunit NDUFB8 was, 64% and 55% lower in JEG3 cells cultured at 1% O_2 than in cells cultured at 21% ($p<0.01$) and 10% O_2 ($p<0.05$), respectively (Figure 1F). The concentration of the complex IV protein subunit (mtCOX2) was also decreased, being 64% and 57% lower in JEG3 cells cultured at 1% O_2 compared with cells cultured at 21% ($p<0.01$) and 10% O_2 ($p<0.01$), respectively (Figure 1F). Since the mRNA expression of these genes was unchanged (Figure 1D), the reduction in the levels of these proteins likely reflects post-transcriptional regulation. Additionally, protein levels of complex III subunit (sub core 2) were 27% and 26% lower in JEG3 cells cultured at 1% O_2 than in control cells cultured at 21% ($p<0.05$) and 10% O_2 ($p<0.05$), respectively (Figure 1F); while this trend was not significant in fibroblasts (Figure 1E). Lastly, complex V subunit (ATP-synthase 5a) showed 26% lower levels of protein in JEG3 cells cultured at 1% O_2 compared with cells at 21% O_2 ($p<0.05$) but not different from cells cultured at 10% O_2 (Figure 1F). The complex II subunit (sub 30 KDa) concentration was not different in fibroblasts and JEG3 cells cultured under all conditions (Figures 1E and 1F). In contrast, but in agreement with respirometry data, in BeWo cells, hypoxia resulted in a downregulation of representative subunits of all five mitochondrial complexes (Figure S1B). Altogether, these data suggest that specific components of the ETS complexes are either transcriptionally or post-transcriptionally regulated in response to hypoxia and that this process may occur in a placental cell-type specific manner. To exclude the possibility that a change in mitochondrial content might underlie these observations, we measured mitochondrial DNA content and citrate synthase levels in both fibroblasts and JEG3 cells in all oxygen conditions and found no significant differences (Figures 2A–D).

Expression of microRNA-210 and its Atargets is Altered in Hypoxic Placental Fibroblasts

One method of post-transcriptional regulation involves miRNAs, which are 23 nucleotide RNAs that bind to complementary sequences on mRNA transcripts and cause gene silencing by translational repression or mRNA degradation [35]. Following our finding that complex I and complex IV protein subunits were decreased in both placental cell types in response to hypoxic conditions, we investigated the expression of miRNA-210, a known regulator of these complexes, along with the expression of its targets COX10 (cytochrome *c* oxidase assembly protein; a subunit of complex IV) and ISCU1/2 (iron-sulfur cluster scaffold proteins), to determine whether the miRNA pathway might play a mecha-

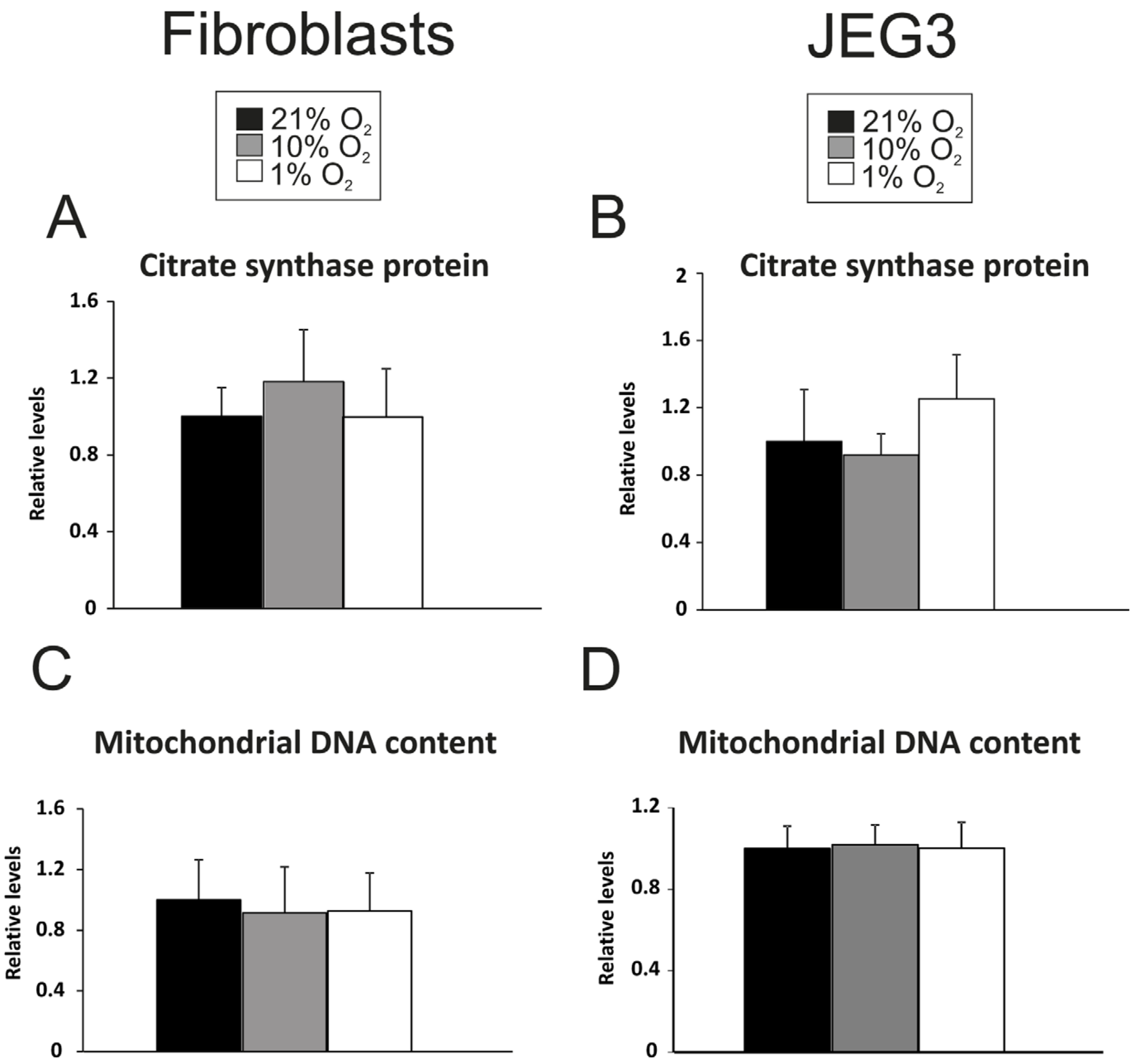

Figure 2. MicroRNA-210 expression is induced in hypoxic fibroblasts but not in hypoxic JEG3 cells; RNA and protein expression of COX10 and ISCU 1/2 are downregulated in both fibroblasts and JEG3 cells cultured in hypoxic condition (1% O_2). ***A, B)*** MiR-210 expression in ***A)*** fibroblasts and in ***B)*** JEG3. ***C, D)*** Transcript levels of COX10 and ISCU1/2 in ***C)*** fibroblasts and *D)* JEG3. ***E, F)*** Protein levels of COX10 and ISCU1/2 in ***E)*** fibroblasts and in ***F)*** JEG3. * $p<0.05$, ** $p<0.01$, *** $p<0.001$ compared with cells cultured at 21% O2; † $p<0.05$ compared with cells cultured at 10% O2. Minimum of three biological replicates per cell type for each condition were performed.
doi:10.1371/journal.pone.0055194.g002

nistic role in this context. Remarkably, exposure of placental fibroblasts to 1% O_2 resulted in a substantial increase in miR-210 levels, 12.8 and 9.7 fold greater than fibroblasts grown at 21% O_2 ($p<0.001$) and 10% O_2 ($p=0.001$), respectively (Figure 3A). This increase in miR-210 in hypoxic fibroblasts also corresponded with a decrease in mRNA and protein levels of its targets COX10 ($p=0.07$; 1% O_2 vs. 10% O_2) and ISCU1/2 ($p<0.01$; 1% O_2 vs. both 10% O_2 and 21% O_2) (Figures 3C, 3E).

Alternatively, in JEG3 cells, there was no change in miR-210 expression under hypoxic conditions (Figure 3B). Yet, we observed an increase in mRNA levels of both *COX10* ($p<0.01$, 1% O_2 vs. 21% O_2; $p<0.05$, 1% O_2 vs. 10% O_2) and *ISCU1/2* ($p<0.05$, 1% O_2 vs. both 10% O_2 and 21% O_2) in hypoxic JEG3 cells (Figure 3D). Despite this increase in mRNA expression, protein levels of COX10 and ISCU1/2 were significantly lower in JEG3 cells exposed to 1% O_2 compared to those at 21% O_2 ($p<0.01$ and $p<0.05$, respectively). These values were also lower than in cells grown at 10% ($p<0.05$; Figure 3F). Altogether, these findings suggest that different placental cell types utilise distinct mechanisms to regulate mitochondrial function: hypoxic fibroblasts initiate a miR-210-based process whereas trophoblast-like JEG3 cells may use an alternative mechanism.

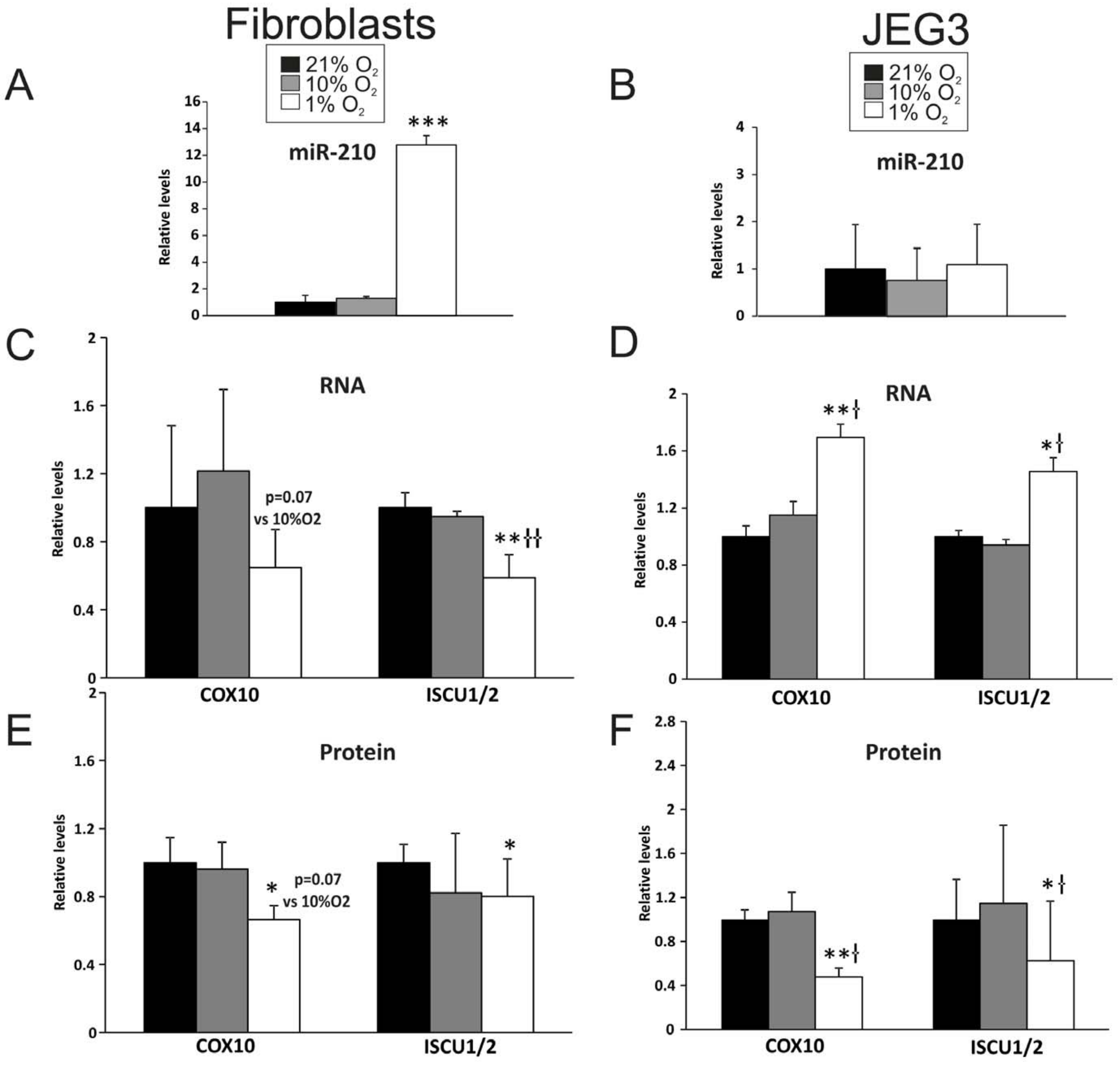

Figure 3. Sublethal dosage of salubrinal downregulates ETS, COX10 and ISCU1/2 protein levels in fibroblasts and JEG3 cells. ***A, B)*** Protein levels of ETS complexes I-IV and V (ATP-synthase) in ***A)*** fibroblasts and ***B)*** JEG3. ***C, D)*** Protein levels of COX10 and ISCU1/2 in ***C)*** fibroblasts and in ***D)*** JEG3. * $p<0.05$, ** $p<0.01$, *** $p<0.001$ compared with cells cultured without salubrinal. Minimum of three biological replicates per cell type for each condition were performed.

doi:10.1371/journal.pone.0055194.g003

Protein Synthesis Inhibition Results in Lower Levels of ETS Proteins in Placental Cells

NDUFB8 transcript levels did not change (Figures 1C and 1D) however protein levels of NDUFB8 were over 60% lower in both cell types under hypoxia, indicating that other mechanism(s) regulate the protein expression. To investigate this further, we cultured primary placental fibroblasts and JEG3 cells in normoxia in the presence and absence of 17.5 μM salubrinal, an inhibitor of the protein synthesis initiation factor eIF2α. Salubrinal did not alter protein levels of representative subunits from ETS complexes II (sub 30 KDa), III (sub core 2) and V (ATP-synthase) in primary placental fibroblasts. However, complex I (NDUFB8) and complex IV (mtCOX2) protein expression was depressed by 88% ($p = 0.06$) and 71% ($p<0.01$), respectively, in fibroblasts cultured with salubrinal relative to those cultured without salubrinal (Figure 4A).

Interestingly, a more robust change was observed in JEG3 cells cultured with salubrinal including a decrease in protein expression of all ETS complexes assessed. Similar to placental fibroblasts, protein levels of complexes I (NDUFB8) and IV (mtCOX2) were 97% and 75% lower in JEG3 cells than in controls ($p<0.0001$ and $p<0.001$, respectively) (Figure 3B). However, analysis of complexes II (sub 30 KDa), III (sub core 2) and V (ATP5a) also revealed a significant repression of protein expression in JEG3 cells

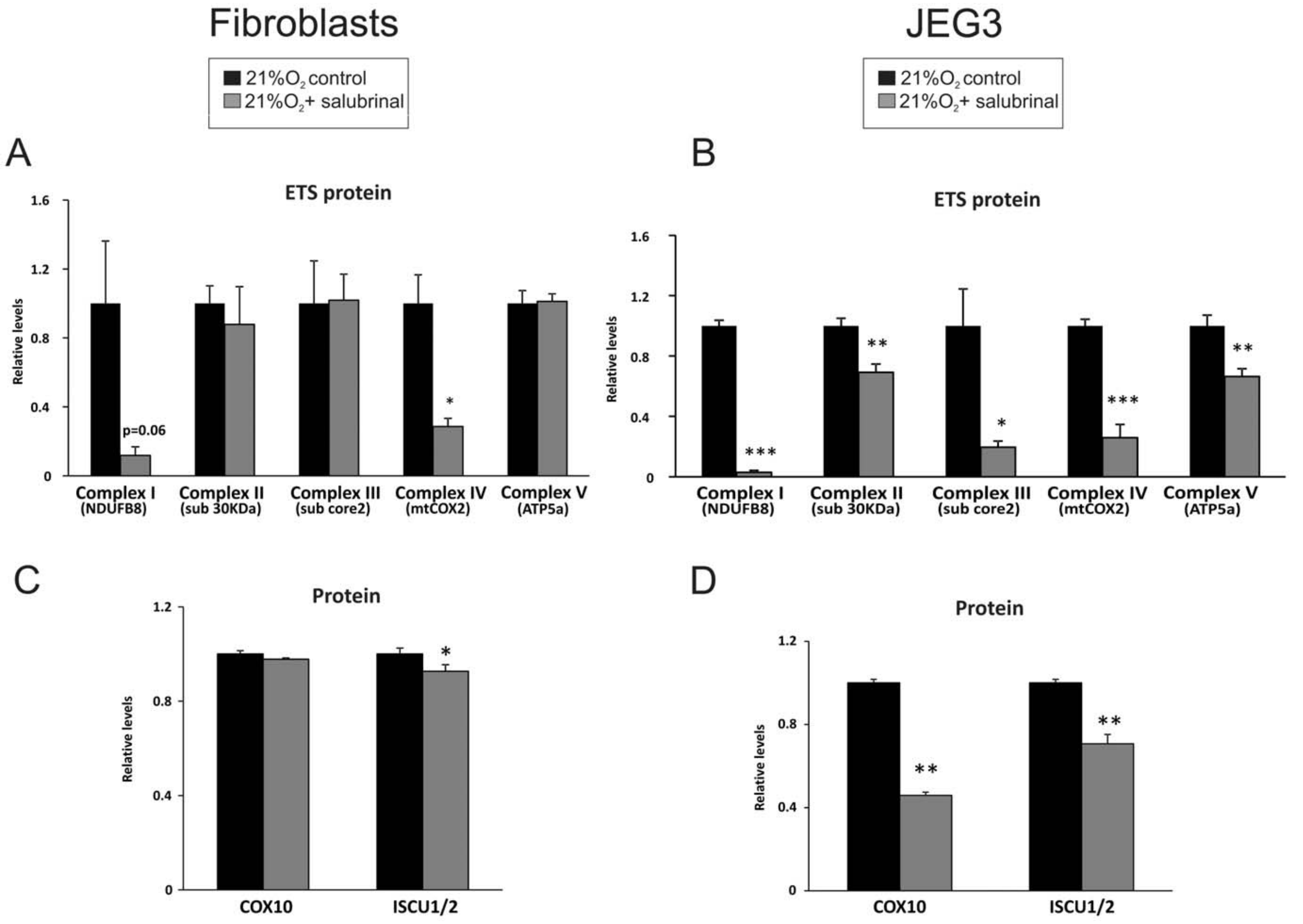

Figure 4. Citrate synthase protein levels and mitochondrial DNA copy number in placental fibroblasts and JEG3 cells. ***A, B)*** Protein levels of citrate synthase in ***A)*** fibroblasts and *B)* JEG3 cells. ***C,D)*** Mitochondrial DNA content in ***C)*** fibroblasts and ***D)*** JEG3. Minimum of four biological replicates per cell type for each condition were performed.
doi:10.1371/journal.pone.0055194.g004

compared to controls by 31% ($p<0.01$), 80% ($p<0.05$) and 34% ($p<0.01$), respectively (Figure 4B).

To further support the idea that there is a link between protein synthesis inhibition and levels of the ETS complexes, we carried out supporting experiments on BeWo cells using other inducers of ER stress: tunicamicin and thapsigargin. Tunicamycin induces the unfolded-protein response and thapsigargin elevates cytosolic Ca^{2+}. Both molecules therefore induce ER stress, via different mechanisms, and in BeWo cells, both inhibitors altered protein levels of representative subunits from ETS complexes (Figure S2A).

We also assessed whether salubrinal affected the expression of COX10 and ISCU1/2 in fibroblasts and JEG3 cells. Salubrinal treatment did not alter COX10 protein levels in fibroblasts (Figure 4C), but levels were 55% lower in JEG3 cells ($p<0.01$) (Figure 3D). Salubrinal decreased ISCU1/2 protein levels by 7% in placental fibroblasts ($p<0.05$) and 30% in JEG3 cells ($p<0.01$) (Figures 4C and 4D). Taken together, these data indicate that protein synthesis inhibition alone can have similar effects to hypoxia, in changing the expression of key proteins required for ETS function. However, placental fibroblast cells appear to be more resistant to the effects of salubrinal and hypoxia than trophoblast-like cells.

High-altitude Pregnancy is Associated with Increased Expression of miR-210

As our *in vitro* data suggests protein synthesis inhibition and miR-210-driven mechanisms likely regulate mitochondrial function in hypoxia, therefore we examined whether similar effects occurred in vivo using placentas from high-altitude pregnancies in which a high level of phosphorylation of eIF2α has been recently identified [28]. Sea-level and high-altitude placentas used in this investigation were matched for maternal age and gestational age and were non-labored, caesarean deliveries. Birth weights were 364 g lower at high altitude than at sea level, and similar to other studies with a trend towards smaller placental weights (Table 1).

There were no differences in mRNA transcript levels of *NDUFB8* (complex I), *mtCOX2* (complex IV) and *ATP5A* (complex V) in high-altitude placentas compared with sea-level controls as revealed by qPCR analysis (Figure–5A). Despite this, protein levels of NDUFB8 were 48% lower in high-altitude placentas than in sea-level placentas ($p<0.01$). Furthermore, protein expression of complexes II (sub 30 KDa), III (sub core 2) and IV (mtCOX2) were lower in high-altitude placentas than in those from sea-level pregnancies, by 36% ($p<0.01$), 28% ($p<0.01$) and 32% ($p<0.05$), respectively. Protein levels of complex V (ATP5a) were, however, unchanged (Figure 5B). These observations are reminiscent of the changes seen in salubrinal-treated JEG3 cells suggesting that the

Table 1. Clinical characteristics of sea-level and high-altitude pregnancies; placental samples from non-labored, caesarean deliveries were used for ETS complex protein, mRNA and microRNA analyses in this study.

	Placentas from Non-Labored Caesarean Deliveries	
	**Sea-Level (n = 6)**	**High-Altitude (n = 3)**
Maternal Age (yrs)	31.5±2.1	34.0±1.0
Gestational Age (wks)	39.0±1.4	39.9±1.2
Birth Weight (g)	3627±272	3263±502
Placental Weight at Birth (g)	545±44	530±35

doi:10.1371/journal.pone.0055194.t001

protein synthesis inhibition, previously reported in these placentas [28] may be acting to suppress mitochondrial respiration.

The expression of miR-210 was 1.8-fold higher in placental samples from high-altitude pregnancies compared to sea-level placentas (p = 0.06; Figure 5D), whilst the mRNA expression of its downstream target gene *COX10* was 27% lower ($p<0.01$), and COX10 protein expression was 30% lower ($p<0.05$) (Figure 5E and 5F). No difference was observed in *ISCU1/2* mRNA expression between the two types of placentas assessed (Figure 5E). However, protein expression of ISCU1/2 was 21% lower (p = 0.06), (Figure 5F), suggesting that miR-210 is likely involved in post-transcriptional repression of ISCU1/2. Overall, these data point to overlapping mechanisms that modify the translational regulation of ETS proteins in high altitude placentas and in placental cells cultured under hypoxic conditions.

Discussion

In all metabolically-active tissues, sustained hypoxia necessitates appropriate responses to maintain energetic and redox homeostasis, and thereby supporting normal cellular function [16]. The hypoxic placenta, however, faces the unique challenge of sustaining sufficient oxygen transfer to the circulation of the developing fetus whilst meeting the oxygen demands of its own

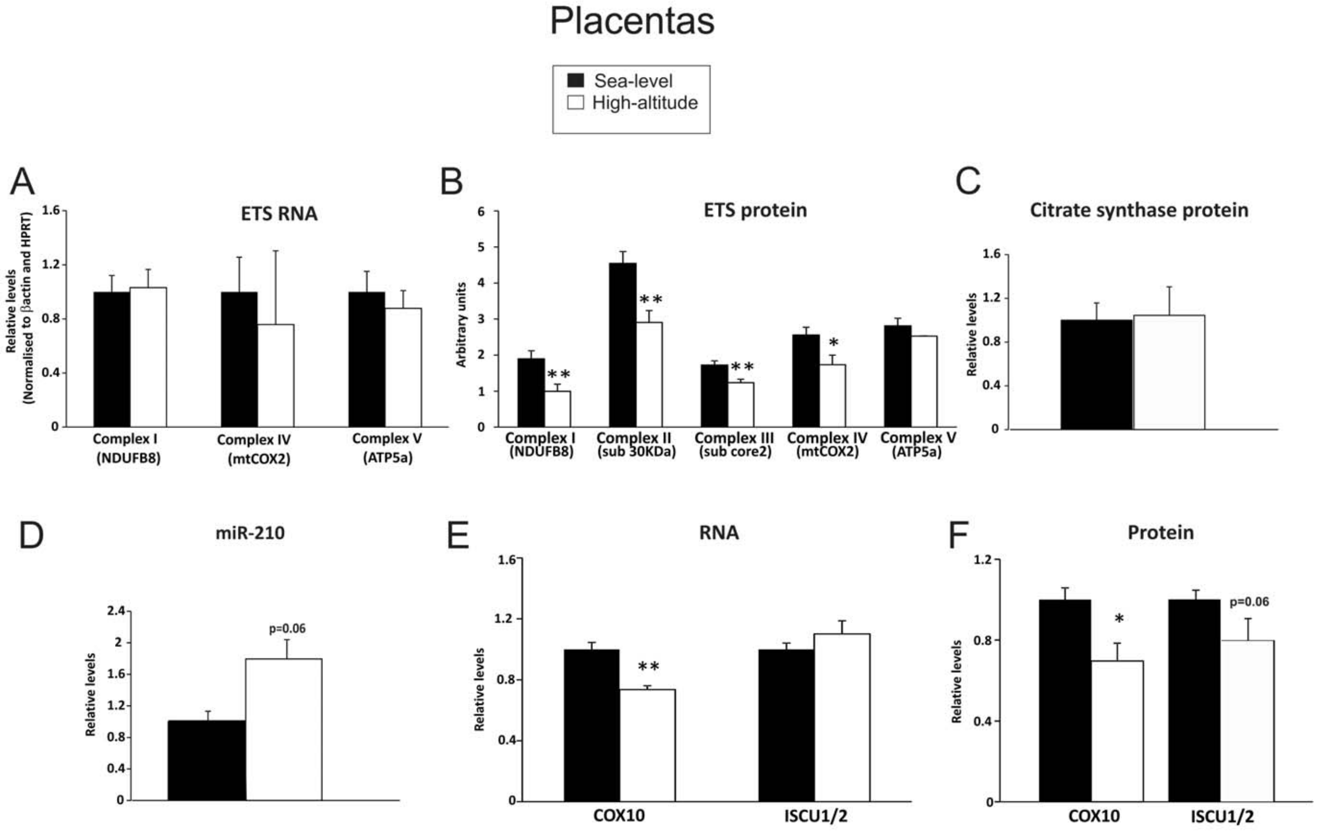

Figure 5. Induction of miR-210, downregulation of ETS, COX10 and ISCU 1/2 protein levels of high-altitude human placentas. ***A)*** Transcript levels of ETS complexes I, IV and V (ATP-synthase) and ***B)*** protein levels of ETS complexes I-IV and V (ATP-synthase). ***C)*** MiR-210 expression in sea-level and high-altitude placentas. ***D)*** Transcript levels and ***E)*** protein levels of COX10 and ISCU1/2. ***F)*** Protein levels of citrate synthase.* $p<0.05$ compared with sea-level placentas. Placentas (n = 6) at sea level (two tissue samples from different regions of each placenta), and 3 placentas at 3100 m altitude (four tissue samples from different regions of each placenta) were performed. Each sample was carried out at least in duplicate.
doi:10.1371/journal.pone.0055194.g005

metabolism [9]. The consequences of a dysfunctional response could either result in fetal oxygen deprivation, if placental oxygen consumption remained too high, or fetal nutrient deprivation, if energetic impairment in the placenta limited the active transport of substrates. A careful partitioning of oxygen between these competing demands is therefore vital to prevent fetal growth restriction and limit oxidative damage in placental and fetal tissues.

We previously found that the high-altitude human placenta is characterised by elevated levels of antioxidant molecules and a lower ATP/ADP ratio, suggesting a diminished energy reserve compared with the sea-level placenta [28]. We therefore hypothesised that altered mitochondrial function plays a role in the placental response to hypoxia and investigated the effects of chronic hypoxia on mitochondrial energy metabolism in human placental cells and the high-altitude placenta. We found a specific decrease in the mitochondrial oxidative capacity of both JEG3 cells and fibroblasts in hypoxia with the ETS complex I substrates, glutamate and malate. Conversely, respiration supported by the complex II substrate, succinate, was unaffected by hypoxia. Complex I protein levels were also lower in both hypoxic fibroblasts and JEG3 cells; though notably transcript levels of NDUF8, a subunit of complex I, were normal in all models, suggesting that a post-translational mechanism underlies this defect. Complex II protein levels were unaffected by hypoxia in cultures, in agreement with normal respiratory rates with succinate.

Complex I (NADH dehydrogenase) is one of the initial complexes of the ETS, and accepts electrons from NADH to reduce ubiquinone. It is, however, a source of the superoxide anion [36], and the decreased activity we report here, which corresponds with findings in hypoxic human skeletal muscle [18], may represent a mechanism to protect mitochondria against oxidative damage. Whilst the major mitochondrial source of ROS in hypoxia is likely to be complex III rather than complex I [37], a decrease in complex I activity could have the effect of suppressing the entry of electrons into the ETS, decreasing ROS production at Complex III [38]. Moreover, in hypoxic JEG3 cells, though not fibroblasts, we also found decreased complex III protein levels. In support of a protective, antioxidant role, we found that mtDNA content was unchanged in both JEG3 cells and fibroblasts cultured at different oxygen concentrations. MtDNA is known to decrease in tissues such as muscle in response to acute oxidative stress and this loss is exacerbated by chronic hypoxia [39]. Moreover, the lack of changes in citrate synthase levels indicates an intrinsic response within mitochondria as opposed to a decrease in mitochondrial mass.

We also recorded decreased respiration rates with the complex IV substrates TMPD and ascorbate in both cell types, suggesting impaired electron transfer to the final acceptor, O_2. Similarly, protein levels of complex IV (cytochrome *c* oxidase) were significantly decreased in both cultures at 1% O_2. Curiously, transcript levels of mtCOX2 (a subunit of complex IV) were unaffected by hypoxia in JEG3 cells, but decreased in fibroblasts, suggesting different regulatory mechanisms. A decreased complex IV activity would serve to match the oxygen demands of placental tissues to the diminished supply, suppressing oxidative metabolism to protect against anoxia-induced cellular injury [40], but perhaps also to allay the risk of fetal oxygen deprivation. Much is known about the regulation of complex IV activity in hypoxic cells, with hypoxia itself restricting O_2 supply to the complex and HIF-dependent upregulation of inducible nitric oxide synthase (iNOS) generating nitric oxide (NO) [41]. NO competes with O_2, thereby reversibly inhibiting complex IV [42], and under sustained levels of NO complex I is also inhibited [43].

Supporting a post-transcriptional regulatory mechanism, we observed a strong upregulation of miR-210 in fibroblasts following hypoxia, perhaps reinforcing the idea of mitochondrial suppression playing a protective role in hypoxia [23,44,45]. No such changes were noted in JEG3 cells, however, though it has been suggested that there are tissue-specific responses of miR-210 in both transformed and primary cell types [46]. Curiously, whilst miR-210 appears to be amongst the most robustly and consistently upregulated micro-RNAs in hypoxia in some carcinoma cell lines [24,46,47], it is downregulated in others [48]. Studies on BeWo cells [47], found an induction of miR-210 after 24h and 48h of incubation at 1% O_2. Since JEG3 cells are metabolically very active, it is possible that they induce miR-210 for a shorter time period than placental fibroblasts, and this might account for the variability seen after 4 d of hypoxic exposure. In support of this, miR-210 was upregulated in isolated trophoblast cells after a short exposure to hypoxia over 8h [27]. Based on these findings, we examined ISCU1/2 and COX10 [24] in the two placental cell types. As expected, we found a downregulation in mRNA and protein levels of both COX10 and ISCU1/2 in placental fibroblasts. In JEG3 cells, hypoxia led to higher transcriptional levels of ISCU1/2 and COX10, but surprisingly the protein levels were drastically lower despite the apparent absence of a miR-210 induction. While these results strengthen the direct correlation between miR-210 and its targets in fibroblasts, an additional mechanism is likely to be at play in JEG3 cells.

Recent published data from our colleagues [28] demonstrated ER stress in high-altitude placental samples. When cultured under the same conditions (1% O2) JEG3 cell proliferation rates were decreased, but fibroblasts were not, Reinforcing the idea of cell-type specific mechanisms, Yung *et al.* showed that hypoxia induced higher levels of eIF2a phosphorylation in JEG3 cells than in fibroblasts [28].

Given these observations, we assessed the effects of a protein synthesis inhibitor (salubrinal) on the levels of ETS complexes and of COX10 and ISCU1/2 *in vitro*. In fibroblasts, levels of both complexes I and IV were lower, reminiscent of the changes in hypoxia. Interestingly, COX10 levels remained unchanged with only a mild reduction in ISCU1/2 levels supporting the idea that miR-210 induction is responsible for these changes in hypoxia. In JEG3 cells, levels of all ETS complexes, plus COX10 and ISCU1/2 were lower than controls, similar to the trend in hypoxia and highlighting protein synthesis inhibition as the major mechanism operating in this cell type. Together, these data indicate that overlapping mechanisms operate in tissue-specific manner in hypoxia, ultimately compromising mitochondrial function, and therefore cellular energetics.

Finally, we investigated whether features of the mechanisms described in our *in vitro* models were present in placentas from high-altitude. Here, we found significant upregulation of miR-210 in high-altitude placentas, which was similar to hypoxic placental fibroblasts, though the increase was milder in comparison. This may be due to the partial contribution of fibroblasts to the whole human placenta, though fibroblasts do account for the largest population of cells in a term placenta, or alternatively to a milder degree of hypoxia *in vivo* compared to the 1% O_2 culture used here. This upregulation correlated with a loss of COX10 and ISCU1/2. Correspondingly, levels of ETS complex subunits were lower in the high-altitude placenta than at sea-level, though this was not reflected in the mRNA levels of these subunits, suggesting a post-translational repression. This may be due to miR-210 upregulation, or protein synthesis inhibition, which Yung *et al.*

[28] found to be present in the same placentas as were studied here. The suppression of mitochondrial respiration in these tissues might necessitate an enhanced flux through glycolytic pathways, and in agreement, we previously reported that these placentas had lower levels of glucose alongside elevated lactate concentrations [49].

The human placenta comprises a number of different tissues, which are functionally, and perhaps metabolically, diverse. Thus it is strength of this study that we have investigated the metabolic response to hypoxia in two distinct placental cell types, as well as in biopsies from human placentas that comprise a heterogeneous cell mix. This study, and our previous metabolomics study of human placentas from high altitude [49], ensures that our *in vitro* findings are relevant to the *in vivo* situation. Moreover, placental metabolism remains a greatly understudied area when compared with other tissues. Unfortunately, due to the damage caused to mitochondrial membranes by freeze-thawing of tissue, we were unable to measure respiration in the placental biopsies from high altitude. A novel cryopreservation technique, recently developed in our laboratory, that must be deployed at the time of collection will allow these measures to be made in future studies [50]. It is also worth noting that due to the logistical difficulties of collecting these samples and the low number of deliveries at Leadville, the number of healthy, non-labored placentas delivered by caesarean section available to us for this study was low. We cannot, therefore, exclude the possibility that we have made a type 2 error in our analyses of ETS complex levels. Indeed, the birth weights of this group were not significantly lower than in the sea-level controls, yet in the larger group of subjects which included labored and non-labored placental samples this did reach significance, in agreement with other studies [3,51].

In conclusion, the human placenta responds to sustained hypoxia by suppressing its own oxygen consumption at the mitochondrial ETS. This might therefore sustain sufficient oxygen transfer to the circulation of the developing fetus. The consequences of this response, however, might underlie the low birth weights of babies at altitude, either by compromising placental energetics and hence transport function, or increasing placental reliance on glycolytic ATP synthesis, resulting in fetal hypoglycaemia, as has been suggested [7]. Similar mechanisms to those we have reported, might underlie some common complications of pregnancy, such as pre-eclampsia and IUGR, where hypoxia and oxidative stress are features of the pathophysiology.

Acknowledgments

We wish to thank the obstetrical nurses of St. Vincent's General Hospital, Leadville, CO, and University College Hospital, London for their co-operation in attaining these samples. We would also like to thank David Menassa and Tom Ashmore for technical assistance, and Prof. Ashley Moffett and Lucy Gardner for FACS analysis.

Supporting Information

Figure S1 Mitochondrial respiratory function and ETS protein expression were altered in BeWo cells cultured in hypoxic conditions. ***A)*** State 2 and state 3 respiration rates with the complex I substrates, glutamate and malate; and state 3 respiration rates with the complex II substrate, succinate, and complex IV substrates, TMPD and ascorbate in BeWo cells. ***B)*** Protein levels of ETS complexes I–IV and V (ATP-synthase) in BeWo cells. Three independent experiments were performed in duplicate for each condition; *$p<0.05$, **$p<0.01$, ***$p<0.001$ compared with cells cultured at 21% O_2.
(TIF)

Figure S2 Treatment with a sublethal dose of tunicamycin (2.5 μg/ml) or thapsigargin (0.4 μM) downregulates ETS protein levels in BeWo cells. ***A)*** Protein levels of ETS complexes I–IV and V (ATP-synthase) in BeWo cells. *$p<0.05$, **$p<0.01$ compared with cells cultured without tunicamycin or thapsigargin. Three biological replicates for control and treated cells were performed.
(TIF)

Author Contributions

Conceived and designed the experiments: FC NP HY EDW IC MCTvP GJB AJM. Performed the experiments: FC NP MCTvP. Analyzed the data: FC NP AJM. Wrote the paper: FC NP HY EDW IC MCTvP GJB AJM.

References

1. Burton GJ (2009) Oxygen, the Janus gas; its effects on human placental development and function. Journal of Anatomy 215: 27–35.
2. Grocott MP, Martin DS, Levett DZ, McMorrow R, Windsor J, et al. (2009) Arterial blood gases and oxygen content in climbers on Mount Everest. N Engl J Med 360: 140–149.
3. Giussani DA, Phillips PS, Anstee S, Barker DJ (2001) Effects of altitude versus economic status on birth weight and body shape at birth. Pediatr Res 49: 490–494.
4. Keyes LE, Armaza JF, Niermeyer S, Vargas E, Young DA, et al. (2003) Intrauterine growth restriction, preeclampsia, and intrauterine mortality at high altitude in Bolivia. Pediatr Res 54: 20–25.
5. Palmer SK, Moore LG, Young D, Cregger B, Berman JC, et al. (1999) Altered blood pressure course during normal pregnancy and increased preeclampsia at high altitude (3100 meters) in Colorado. Am J Obstet Gynecol 180: 1161–1168.
6. Postigo L, Heredia G, Illsley NP, Torricos T, Dolan C, et al. (2009) Where the O2 goes to: preservation of human fetal oxygen delivery and consumption at high altitude. J Physiol 587: 693–708.
7. Zamudio S, Torricos T, Fik E, Oyala M, Echalar L, et al. (2010) Hypoglycemia and the origin of hypoxia-induced reduction in human fetal growth. PLoS One 5: e8551.
8. Illsley NP, Caniggia I, Zamudio S (2010) Placental metabolic reprogramming: do changes in the mix of energy-generating substrates modulate fetal growth? The International Journal of Developmental Biology 54: 409–419.
9. Murray AJ (2012) Oxygen delivery and fetal placental growth: beyond a question of supply and demand? Placenta Suppl 2: e16–e22.
10. Rennie JM (2002) A review of the risk of being born too soon. Fetal and Maternal Medicine Review 13: 157–168.
11. Redman CW, Sargent IL (2005) Latest advances in understanding preeclampsia. Science 308: 1592–1594.
12. Hung TH, Burton GJ (2006) Hypoxia and reoxygenation: a possible mechanism for placental oxidative stress in preeclampsia. Taiwan J Obstet Gynecol 45: 189–200.
13. Gandley RE, Rohland J, Zhou Y, Shibata E, Harger GF, et al. (2008) Increased myeloperoxidase in the placenta and circulation of women with preeclampsia. Hypertension 52: 387–393.
14. Many A, Hubel CA, Fisher SJ, Roberts JM, Zhou Y (2000) Invasive cytotrophoblasts manifest evidence of oxidative stress in preeclampsia. American Journal of Pathology 156: 321–331.
15. Wang Y, Walsh SW (1998) Placental mitochondria as a source of oxidative stress in pre-eclampsia. Placenta 19: 581–586.
16. Murray AJ (2009) Metabolic adaptation of skeletal muscle to high altitude hypoxia: how new technologies could resolve the controversies. Genome Med 1: 117.
17. Murray AJ, Edwards LM, Clarke K (2007) Mitochondria and heart failure. Curr Opin Clin Nutr Metab Care 10: 704–711.
18. Levett DZ, Radford EJ, Menassa DA, Graber EF, Morash AJ, et al. (2012) Acclimatization of skeletal muscle mitochondria to high-altitude hypoxia during an ascent of Everest. Faseb Journal 26: 1431–1441.
19. Burton GJ, Yung HW (2011) Endoplasmic reticulum stress in the pathogenesis of early-onset pre-eclampsia. Pregnancy Hypertens 1: 72–78.
20. Papandreou I, Cairns RA, Fontana L, Lim AL, Denko NC (2006) HIF-1 mediates adaptation to hypoxia by actively downregulating mitochondrial oxygen consumption. Cell Metabolism 3: 187–197.
21. Simon MC (2006) Coming up for air: HIF-1 and mitochondrial oxygen consumption. Cell Metabolism 3: 150–151.

22. Kulshreshtha R, Ferracin M, Wojcik SE, Garzon R, Alder H, et al. (2007) A microRNA signature of hypoxia. Mol Cell Biol 27: 1859–1867.
23. Chan SY, Zhang YY, Hemann C, Mahoney CE, Zweier JL, et al. (2009) MicroRNA-210 controls mitochondrial metabolism during hypoxia by repressing the iron-sulfur cluster assembly proteins ISCU1/2. Cell Metab 10: 273–284.
24. Chen Z, Li Y, Zhang H, Huang P, Luthra R (2010) Hypoxia-regulated microRNA-210 modulates mitochondrial function and decreases ISCU and COX10 expression. Oncogene 29: 4362–4368.
25. Zhu XM, Han T, Sargent IL, Yin GW, Yao YQ (2009) Differential expression profile of microRNAs in human placentas from preeclamptic pregnancies vs normal pregnancies. Am J Obstet Gynecol 200: 661 e661–667.
26. Mouillet J-F, Chu T, Sadovsky Y (2011) Expression patterns of placental microRNAs. Birth Defects Research Part A: Clinical and Molecular Teratology 91: 737–743.
27. Muralimanoharan S, Maloyan A, Mele J, Guo C, Myatt LG, et al. (2012) MIR-210 modulates mitochondrial respiration in placenta with preeclampsia. Placenta 33: 816–823.
28. Yung HW, Cox M, Tissot van Patot M, Burton GJ (2012) Evidence of endoplasmic reticulum stress and protein synthesis inhibition in the placenta of non-native women at high altitude. FASEB J 26: 1970–1981.
29. Matsuo H, Strauss JF 3rd (1994) Peroxisome proliferators and retinoids affect JEG-3 choriocarcinoma cell function. Endocrinology 135: 1135–1145.
30. Apps R, Sharkey A, Gardner L, Male V, Trotter M, et al. (2011) Genome-wide expression profile of first trimester villous and extravillous human trophoblast cells. Placenta 32: 33–43.
31. Cindrova-Davies T, Yung HW, Johns J, Spasic-Boskovic O, Korolchuk S, et al. (2007) Oxidative stress, gene expression, and protein changes induced in the human placenta during labor. Am J Pathol 171: 1168–1179.
32. Kuznetsov AV, Veksler V, Gellerich FN, Saks V, Margreiter R, et al. (2008) Analysis of mitochondrial function in situ in permeabilized muscle fibers, tissues and cells. Nat Protoc 3: 965–976.
33. Heather LC, Cole MA, Lygate CA, Evans RD, Stuckey DJ, et al. (2006) Fatty acid transporter levels and palmitate oxidation rate correlate with ejection fraction in the infarcted rat heart. Cardiovasc Res 72: 430–437.
34. Tannetta DS, Sargent IL, Linton EA, Redman CW (2008) Vitamins C and E inhibit apoptosis of cultured human term placenta trophoblast. Placenta 29: 680–690.
35. Bartel DP (2009) MicroRNAs: target recognition and regulatory functions. Cell 136: 215–233.
36. Turrens JF, Boveris A (1980) Generation of superoxide anion by the NADH dehydrogenase of bovine heart mitochondria. Biochem J 191: 421–427.
37. Guzy RD, Schumacker PT (2006) Oxygen sensing by mitochondria at complex III: the paradox of increased reactive oxygen species during hypoxia. Exp Physiol 91: 807–819.
38. Wheaton WW, Chandel NS (2011) Hypoxia. 2. Hypoxia regulates cellular metabolism. Am J Physiol Cell Physiol 300: C385–393.
39. Puente-Maestu L, Lazaro A, Tejedor A, Camano S, Fuentes M, et al. (2010) Effects of exercise on mitochondrial DNA content in skeletal muscle of patients with COPD. Thorax 66: 121–127.
40. Hochachka PW (1986) Defense strategies against hypoxia and hypothermia. Science 231: 234–241.
41. Melillo G, Taylor LS, Brooks A, Musso T, Cox GW, et al. (1997) Functional requirement of the hypoxia-responsive element in the activation of the inducible nitric oxide synthase promoter by the iron chelator desferrioxamine. J Biol Chem 272: 12236–12243.
42. Cleeter MW, Cooper JM, Darley-Usmar VM, Moncada S, Schapira AH (1994) Reversible inhibition of cytochrome c oxidase, the terminal enzyme of the mitochondrial respiratory chain, by nitric oxide. Implications for neurodegenerative diseases. FEBS Lett 345: 50–54.
43. Clementi E, Brown GC, Feelisch M, Moncada S (1998) Persistent inhibition of cell respiration by nitric oxide: crucial role of S-nitrosylation of mitochondrial complex I and protective action of glutathione. Proc Natl Acad Sci U S A 95: 7631–7636.
44. Puisségur MP, Mazure NM, Bertero T, Pradelli L, Grosso S, et al. (2010) miR-210 is overexpressed in late stages of lung cancer and mediates mitochondrial alterations associated with modulation of HIF-1 activity. Cell Death and Differentiation 18: 465–478.
45. Kulshreshtha R, Davuluri RV, Calin GA, Ivan M (2008) A microRNA component of the hypoxic response. Cell Death Differ 15: 667–671.
46. Chan SY, Loscalzo J (2010) MicroRNA-210: a unique and pleiotropic hypoxamir. Cell Cycle 9: 1072–1083.
47. Lee D-C, Romero R, Kim J-S, Tarca AL, Montenegro D, et al. (2011) miR-210 Targets Iron-Sulfur Cluster Scaffold Homologue in Human Trophoblast Cell Lines. The American Journal of Pathology 179: 590–602.
48. Giannakakis A, Sandaltzopoulos R, Greshock J, Liang S, Huang J, et al. (2008) miR-210 links hypoxia with cell cycle regulation and is deleted in human epithelial ovarian cancer. Cancer Biol Ther 7: 255–264.
49. Tissot van Patot MC, Murray AJ, Beckey V, Cindrova-Davies T, Johns J, et al. (2010) Human placental metabolic adaptation to chronic hypoxia, high altitude: hypoxic preconditioning. Am J Physiol Regul Integr Comp Physiol 298: R166–172.
50. Colleoni F, Morash AJ, Ashmore T, Monk M, Burton GJ, et al. (2012) Cryopreservation of placental biopsies for mitochondrial respiratory analysis. Placenta 33: 122–123.
51. Moore LG (2003) Fetal growth restriction and maternal oxygen transport during high altitude pregnancy. High Alt Med Biol 4: 141–156.
